# Editorial for the Special Issue “Novel Echocardiographic Techniques for the Assessment of Cardiovascular Disease”

**DOI:** 10.3390/medicina61020345

**Published:** 2025-02-15

**Authors:** Konstantinos Katogiannis

**Affiliations:** 2nd Cardiology Department, National and Kapodistrian University of Athens, General Hospital “Attikon”, 12462 Chaidari, Attiki, Greece; kenndj89@gmail.com

The global burden of cardiovascular disease (CVD) continues to pose a significant challenge to healthcare systems worldwide. Despite advances in prevention, diagnosis, and management, CVD remains the leading cause of morbidity and mortality globally. Against this backdrop, echocardiography has emerged as one of the most pivotal tools in cardiovascular medicine. This non-invasive imaging modality has evolved dramatically since its inception, with innovations that provide unparalleled insights into cardiac structure, function, and hemodynamics. This Special Issue of *Medicina*, titled “Novel Echocardiographic Techniques for the Assessment of Cardiovascular Disease,” serves as a platform to explore groundbreaking advancements in this field, highlighting their potential to revolutionize clinical practice.

Echocardiography has undergone remarkable transformations over the past decades, transitioning from M-mode and two-dimensional imaging to three-dimensional echocardiography (3DE), strain imaging, contrast-enhanced techniques, and artificial intelligence (AI)-driven analytics. These developments have expanded the scope of echocardiography beyond the traditional diagnostic applications to encompass risk stratification, therapy monitoring, and prognostication.

Beyond conventional 2D imaging, 3DE provides volumetric data in real-time, enabling the precise assessment of cardiac chambers and valve morphology. Its utility in pre-procedural planning for interventions like transcatheter aortic valve replacement (TAVR) has been transformative [1].

Moreover, speckle-tracking echocardiography (STE) has emerged as a cornerstone for assessing myocardial mechanics. Global longitudinal strain (GLS), a parameter derived from STE, has demonstrated superior sensitivity in detecting subclinical myocardial dysfunctions, particularly in conditions like heart failure with preserved ejection fraction (HFpEF) and chemotherapy-induced cardiotoxicity [2].

Further, the use of contrast agents has significantly improved the visualization of cardiac structures and perfusions. CEE is particularly valuable for detecting intracardiac thrombi, assessing myocardial viability, and evaluating coronary microvascular functioning [3].

Artificial intelligence (AI) and machine learning (ML) are reshaping the echocardiographic landscape. Automated image acquisitions and interpretations promise to enhance reproducibility, reduce operator dependency, and streamline workflows. AI-driven algorithms can detect subtle abnormalities that may elude conventional analyses, thereby improving diagnostic accuracy and patient outcomes [4].

For example, AI-enhanced strain analysis and automated left ventricular ejection fraction (LVEF) quantification have shown great promise in both research and clinical settings. Moreover, AI’s role in risk prediction and decision support is rapidly expanding, offering tailored therapeutic strategies for patients with CVD [5].

The ability to accurately evaluate cardiac functions and structures is central to managing CVD. This Special Issue highlights innovations in functional imaging. Novel echocardiographic parameters, including left atrial strain and diastolic strain rate, provide deeper insights into left ventricular diastolic dysfunction, a key driver of HFpEF [6]. Advancements in 3D transesophageal echocardiography (TEE) have improved the quantification of mitral regurgitation and aortic stenosis severity. Additionally, fusion imaging techniques combining echocardiography with computed tomography (CT) enhance procedural success rates in structural heart interventions [7]. Emerging techniques, such as myocardial work analysis, integrate pressure-strain loops to assess myocardial efficiency, offering an incremental value over traditional measures in CAD evaluation [8].

The clinical implications of these novel echocardiographic techniques are profound. They facilitate early detection of subclinical disease, allowing for timely interventions and improved outcomes. Further, treatment strategies are tailored based on detailed echocardiographic data, and risk stratification models can predict adverse outcomes more accurately.

For instance, GLS has proven to be a superior prognostic marker in patients undergoing cancer therapy, providing critical insights into the risk of cardiotoxicity and enabling proactive management [9].

Moreover, left atrial strain (LAS) analysis using STE is rapidly emerging as a critical tool in diagnosing and prognosticating cardiovascular pathologies. A recent review by O’Neill et al. highlights LAS’s capacity to detect subtle functional impairments before morphological changes occur, making it an earlier marker for diseases like heart failure and atrial fibrillation. LAS is divided into three phases—reservoir, conduit, and booster strain—each corresponding to distinct phases of the cardiac cycle. Its role in predicting outcomes in atrial cardiomyopathy, heart failure with preserved ejection fraction (HFpEF), and even thromboembolic risk stratification, is promising [10].

For example, the LAS reservoir strain (LASr) has shown remarkable utility in the early detection of diastolic dysfunctions, even in patients without left atrial enlargement. The reservoir phase, capturing LA filling during systole, is often the first to deteriorate in cardiovascular diseases. Its sensitivity to early dysfunction makes LAS an invaluable addition to standard echocardiography. The reliability and accessibility of STE ensures its potential for widespread clinical use, provided that further studies validate its routine implementation [10].

Building on the understanding of LAS, Bingöl et al. explored its sensitivity to subclinical cardiac dysfunction in patients with impaired fasting glucose (IFG). IFG, a precursor to diabetes mellitus (DM), is associated with increased cardiovascular risk, even before the onset of overt DM. Their study demonstrated significant reductions in LAS parameters, such as reservoir strain and conduit strain, in individuals with IFG compared to normoglycemic counterparts [11].

This finding underscores the early involvement of the left atrial function in metabolic disturbances. LAS could thus serve as a surrogate marker for identifying high-risk individuals and tailoring interventions to prevent progression to diabetes and its cardiovascular complications. Integrating LAS assessment into routine echocardiographic protocols for patients with prediabetes might bridge the gap between diagnosis and early intervention, improving long-term cardiovascular outcomes.

The study by Katogiannis et al. [12] investigated the effects of liraglutide, empagliflozin, and their combination on left atrial (LA) function and arterial stiffness in patients with type 2 diabetes mellitus (T2DM). Over a six-month period, the researchers assessed changes in LA strain parameters and arterial function among patients treated with these medications compared to those receiving insulin therapy.

Their findings suggest that treatment with liraglutide, empagliflozin, and their combination leads to greater improvements in LA function and reductions in arterial stiffness compared to insulin therapy in patients with T2DM. These results highlight the potential cardiovascular benefits of these medications beyond glycemic control.

Furthermore, in the context of advanced heart failure, heart transplantation remains the definitive therapeutic option for selected patients. Postoperative management and prognostication are critical, and the study by Huma et al. introduces the TAPSE/sPAP ratio as a robust, cost-effective marker for assessing right ventricular (RV) function and predicting short-term survival [13].

The TAPSE/sPAP ratio reflects RV-PA coupling, a crucial determinant of hemodynamic stability in transplant recipients. Huma et al. identified a threshold of 0.47 mm/mmHg for predicting 6-month survival, with a strong association between higher ratios and improved outcomes. Importantly, this metric relies on widely accessible echocardiographic data, making it practical for routine clinical use even in resource-limited settings.

Despite these advancements, several challenges remain. High costs, limited access to advanced technologies in resource-limited settings, and the need for extensive training to interpret complex imaging data are significant barriers. Additionally, the integration of AI into clinical practice requires robust validation, addressing concerns related to transparency, bias, and data privacy.

Future research should focus on simplifying Advanced Techniques, Expanding Accessibility and Enhancing Integration.

The contributions to this Special Issue of *Medicina* underscore the transformative potential of novel echocardiographic techniques in the assessment of cardiovascular disease. These innovations not only enhance diagnostic accuracy but also pave the way for personalized and precision medicine. By addressing current challenges and leveraging emerging technologies, the future of echocardiography holds immense promise for improving patient care and outcomes.

We extend our gratitude to the authors, reviewers, and editorial team who have made this Special Issue possible. Their dedication and expertise are instrumental in advancing the field of cardiovascular imaging.

## References

[1] Lang R.M., Badano L.P., Tsang W., Adams D.H., Agricola E., Buck T., Faletra F.F., Franke A., Hung J., de Isla L.P. (2012). EAE/ASE recommendations for image acquisition and display using three-dimensional echocardiography. J. Am. Soc. Echocardiogr..

[2] Thavendiranathan P., Poulin F., Lim K.D., Plana J.C., Woo A., Marwick T.H. (2014). Use of myocardial strain imaging by echocardiography for the early detection of cardiotoxicity in patients during and after cancer chemotherapy: A systematic review. J. Am. Coll. Cardiol..

[3] Senior R., Becher H., Monaghan M., Agati L., Zamorano J., Vanoverschelde J.L., Nihoyannopoulos P. (2009). Contrast echocardiography: Evidence-based recommendations by European Association of Echocardiography. Eur. J. Echocardiogr..

[4] Kusunose K. (2023). Revolution of echocardiographic reporting: The new era of artificial intelligence and natural language processing. J. Echocardiogr..

[5] Voigt J.U., Cvijic M. (2019). 2- and 3-Dimensional Myocardial Strain in Cardiac Health and Disease. JACC Cardiovasc. Imaging.

[6] Frydas A., Morris D.A., Belyavskiy E., Radhakrishnan A.K., Kropf M., Tadic M., Roessig L., Lam C.S.P., Shah S.J., Solomon S.D. (2020). Left atrial strain as sensitive marker of left ventricular diastolic dysfunction in heart failure. ESC Heart Fail..

[7] Zoghbi W.A., Chambers J.B., Dumesnil J.G., Foster E., Gottdiener J.S., Grayburn P.A., Khandheria B.K., Levine R.A., Marx G.R., Miller F.A. (2009). Recommendations for evaluation of prosthetic valves with echocardiography and Doppler ultrasound. J. Am. Soc. Echocardiogr..

[8] Sengupta P.P., Adjeroh D.A. (2018). Will Artificial Intelligence Replace the Human Echocardiographer?. Circulation.

[9] Plana J.C., Galderisi M., Barac A., Ewer M.S., Ky B., Scherrer-Crosbie M., Ganame J., Sebag I.A., Agler D.A., Badano L.P. (2014). Expert consensus for multimodality imaging evaluation of adult patients during and after cancer therapy: A report from the American Society of Echocardiography and the European Association of Cardiovascular Imaging. Eur. Heart J.—Cardiovasc. Imaging.

[10] O’Neill T., Kang P., Hagendorff A., Tayal B. (2024). The Clinical Applications of Left Atrial Strain: A Comprehensive Review. Medicina.

[11] Bingöl G., Avcı Demir F., Özmen E., Ünlü S., Özden Ö., Tokdil K.O., Arsoy L.B., Özpamuk Karadeniz F., Ökçün B. (2023). Effects of an Impaired Fasting Glucose on the Left Atrial Strain Evaluated by Speckle Tracking Echocardiography. Medicina.

[12] Katogiannis K., Thymis J., Kousathana F., Pavlidis G., Korakas E., Kountouri A., Balampanis K., Prentza V., Kostelli G., Michalopoulou H. (2024). Effects of Liraglutide, Empagliflozin and Their Combination on Left Atrial Strain and Arterial Function. Medicina.

[13] Huma L., Suciu H., Avram C., Suteu R.-A., Danilesco A., Baba D.-F., Moldovan D.-A., Sin A.-I. (2024). Tricuspid Annular Plane Systolic Excursion-to-Systolic Pulmonary Artery Pressure Ratio as a Prognostic Factor in Heart Transplant Patients. Medicina.

